# Benign granular cell tumor of the abdominal wall mimicking postpartum desmoid tumor

**DOI:** 10.1007/s00256-025-04913-6

**Published:** 2025-03-25

**Authors:** Wendy Qiu, Zaid Patel, Joshua Pierce, Timothy Iafe

**Affiliations:** 1 Department of Radiological Sciences, UCLA David Geffen School of Medicine, Los Angeles, CA USA; 2 Department of Pathology & Laboratory Medicine, UCLA David Geffen School of Medicine, Los Angeles, CA USA

**Keywords:** Granular cell tumors, Abdominal wall, Rare soft tissue tumors, Schwann cells, Desmoid tumor

## Abstract

Granular cell tumors are uncommon soft tissue neoplasms derived from Schwann cells. It is extremely rare for granular cell tumors to be found in the anterior abdominal wall, with only 12 cases of benign abdominal wall granular cell tumors reported in the medical literature to date. We report a case of an upper abdominal wall granular cell tumor in a recently postpartum 35-year-old woman. Based on the patient’s history, recent postpartum presentation, and imaging findings, the soft tissue tumor was initially suspected to be a desmoid tumor. Therefore, following biopsy and resection, the final histopathological diagnosis of benign granular cell tumor was quite unusual. Herein, we discuss a unique presentation of benign granular cell tumor and its diagnostic workup, including imaging and histopathologic findings, to highlight the possibility of this rare entity in the differential diagnosis of certain abdominal wall soft tissue masses.

## Introduction

Granular cell tumors (GrCT) are rare soft tissue neoplasms derived from Schwann cells that can involve a variety of anatomical sites. It is extremely rare for GrCT to be found in the anterior abdominal wall, and, to our knowledge, only 12 cases of benign abdominal wall GrCT have been reported in the medical literature to date (Table 1) [1–12]. GrCT are frequently benign tumors; however, up to 2% may present with malignant features [13, 14]. On imaging, GrCT appear as well-defined soft tissue masses with variable CT attenuation and MRI signal intensities, and the differential diagnosis includes other neoplasms, such as desmoid tumors and sarcomas. Here, we present a rare case of an upper abdominal wall granular cell tumor in a 35-year-old female who was 3 months postpartum at presentation. This mass clinically resembled an abdominal wall desmoid tumor due to the patient’s recently postpartum history, clinical presentation, and imaging findings. Further, we discuss the diagnostic workup of GrCT including imaging and histopathologic findings, the Fanburg-Smith benign versus malignant classification criteria, and imaging overlap of GrCT with other soft tissue neoplasms such as desmoid tumor.

**Table 1:** Benign GrCTs*

Case	Age	Sex	Clinical presentation	Abdominal wall localization	Imaging findings	Reference
1	43	F	Abdominal wall lump incidentally found at routine clinical examination	Intramuscular; left rectus abdominis muscle	US: 4.1 × 0.8 × 0.9 cm heterogeneous, hypoechoic mass with irregular spiculated posterior margins MRI: 2.6 × 1.7 × 3.4 cm well-defined intramuscular hypointense mass within the left rectus abdominis muscle with rim enhancement	Mangan (2024) [1]
2	73	F	Nontender, mobile subcutaneous mass, firm to hard in consistency	Subcutaneous; right anterior abdominal wall, halfway between inferior costal margin and the iliac crest along the anterior axillary line	US: 0.9 × 0.5 × 1.3 cm hyperechoic solid lesion with posterior shadowing CT: Soft tissue nodule within the anterior abdominal wall subcutaneous tissue	Rehan (2021) [3]
3	60	F	Incidental finding	Intramuscular; left posterolateral abdominal wall muscles	CT: Intramuscular soft tissue mass without significant enhancement MRI: Homogeneous, moderate enhancement FDG PET: Increased FDG uptake at the abdominal wall lesion	Saito (2018) [8]
4	45	F	Pain in the upper third of the right abdominal wall	Intramuscular; right rectus abdominis muscle	US: 2.0 cm oval lesion with inhomogeneous echogenicity and internal vascularity CECT: Inhomogeneous nodule localized in the right rectus abdominis muscle with moderate enhancement MRI: T1-hypointense, T2-hypointense, homogeneously enhancing, without signs of invasion into the underlying peritoneum	Porta (2015) [2]
5	49	F	Nontender, palpable mass in left upper quadrant abdomen	Subcutaneous; left subcostal anterior abdominal wall	US: 2.2 cm slightly ill-marginated hypoechoic mass MRI: Well circumscribed enhancing soft-tissue mass	McGhan (2015) [4]
6	29	F	Subcutaneous mass with hard consistency fixed to the deep muscle layer	Intramuscular; right abdominal wall	US: 1.2 × 0.9 × 1.0 cm well-defined, round, hypoechoic, homogeneous and avascular mass	Panunzi (2012) [9]
7	50	F	Painless, palpable, firm and flat subcutaneous mass at the abdominal wall	Subcutaneous; left lower abdominal wall near umbilicus	No imaging was performed	Wang (2015) [5]
8	70	F	Fixed, firm lump in the left iliac fossa area of the abdomen	Intramuscular; internal oblique and transversus abdominis	CT: Soft tissue mass arising from the left anterior abdominal wall muscles, in particular the internal oblique and transversus abdominis	Chaudhry (2008) [6]
9	44	F	Nontender, hard mass in right lower abdominal wall	Intramuscular; right rectus abdominis muscle	US: Well-defined, hypoechoic mass in the rectus muscle of the abdominal wall CT: 3.6 × 2.5 cm ovoid mass in the right rectus muscle	An (2007) [7]
10	37	M	Nontender, smooth lump on the lower right side of the abdomen, midway between pubis and umbilicus	Intramuscular; right rectus abdominis	US: Solid mass in the right rectus abdominis muscle sheath measuring 2.7 × 2.0 × 1.6 cm	Joshi (2003) [10]

^*^Two additional cases of abdominal wall benign granular cell tumor abstracts were identified; however, full reports were not available online, and therefore, they were excluded from the table [11, 12]

## Case report

A 35-year-old female presented to her gynecologist at 3 months postpartum with a palpable, nontender, right inframammary mass. On physical examination, an approximately 5 cm firm, nontender, solid nodule was noted at the right inframammary body wall along the lower costal margin. No other lumps or masses, erythema, or skin/nipple changes of the right breast were noted. Prior to any diagnostic workup, this mass was clinically suspected to be related to the ribs or a breast lesion, given its location, firmness, and the patient’s ongoing breastfeeding. The patient was subsequently referred to breast radiology for diagnostic breast ultrasound.

**Fig. 1:**
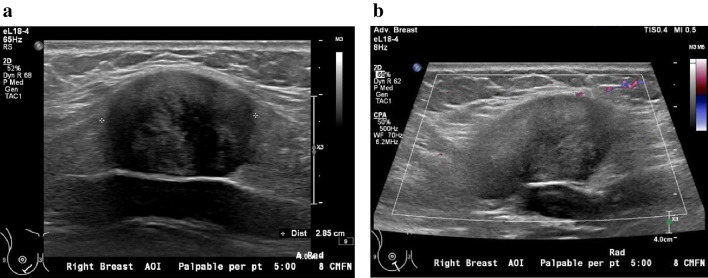
Initial grayscale breast ultrasound (**a**) demonstrates a well-circumscribed heterogeneously hypoechoic soft tissue mass at the thoracoabdominal wall, measuring 3.8 × 2.0 × 2.9 cm. On color Doppler ultrasound (**b**), this mass was avascular. The lesion is deep to and separate from the breast parenchyma

**Fig. 2:**
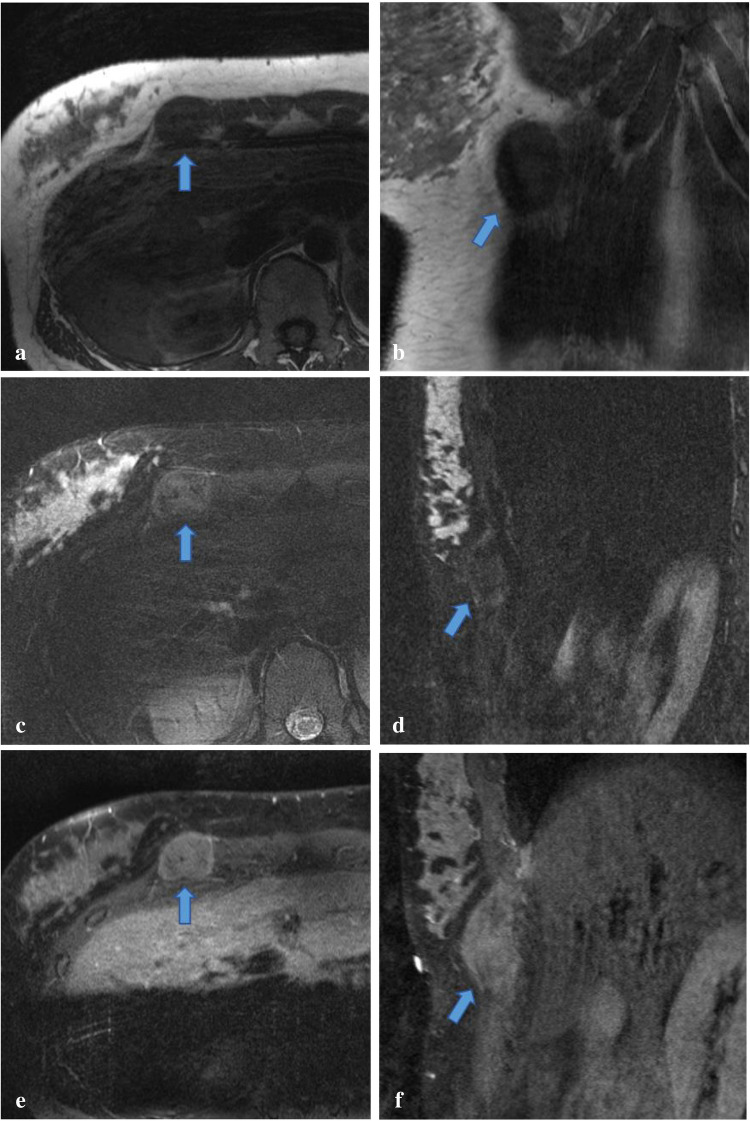
Thirty-five-year-old female with granular cell tumor of the right rectus abdominis muscle. Axial T1 (**a**), coronal T1 (**b**), axial T2 fat-saturated (**c**), sagittal inversion recovery (**d**), postcontrast axial T1 fat-suppressed (**e**), and postcontrast sagittal T1 fat-suppressed (**f**) MR images of the inframammary right chest wall demonstrate a well-circumscribed 3.2 × 2.4 × 4.7 cm mass within the superior and lateral portion of the right rectus abdominis muscle. There is no rib, intercostal, intrathoracic, or breast involvement

Targeted breast ultrasound revealed a well-circumscribed, heterogeneously hypoechoic intramuscular body wall mass located deep to and separate from the right breast parenchyma (Fig. 1). As such, the patient was referred for an MRI of the chest wall, which revealed a well-circumscribed 3.2 × 2.4 × 4.7 cm mass within the superolateral portion of the right rectus abdominis muscle (Fig. 2). MRI features were nonspecific but were suggestive of a non-aggressive intramuscular mass, distinct from the breast parenchyma. Given the lesion’s location and firmness, as well as the patient’s recently postpartum presentation, the presumed pre-biopsy diagnosis was a desmoid tumor.

**Fig. 3:**
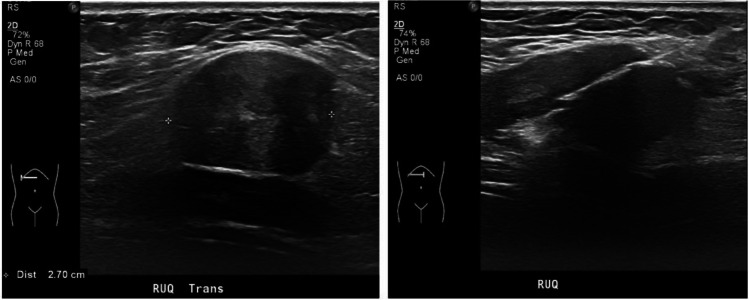
Ultrasound biopsy of the right upper abdominal wall mass. The mass was perceived as very firm or dense, providing high resistance to penetration with the biopsy needle

**Fig. 4:**
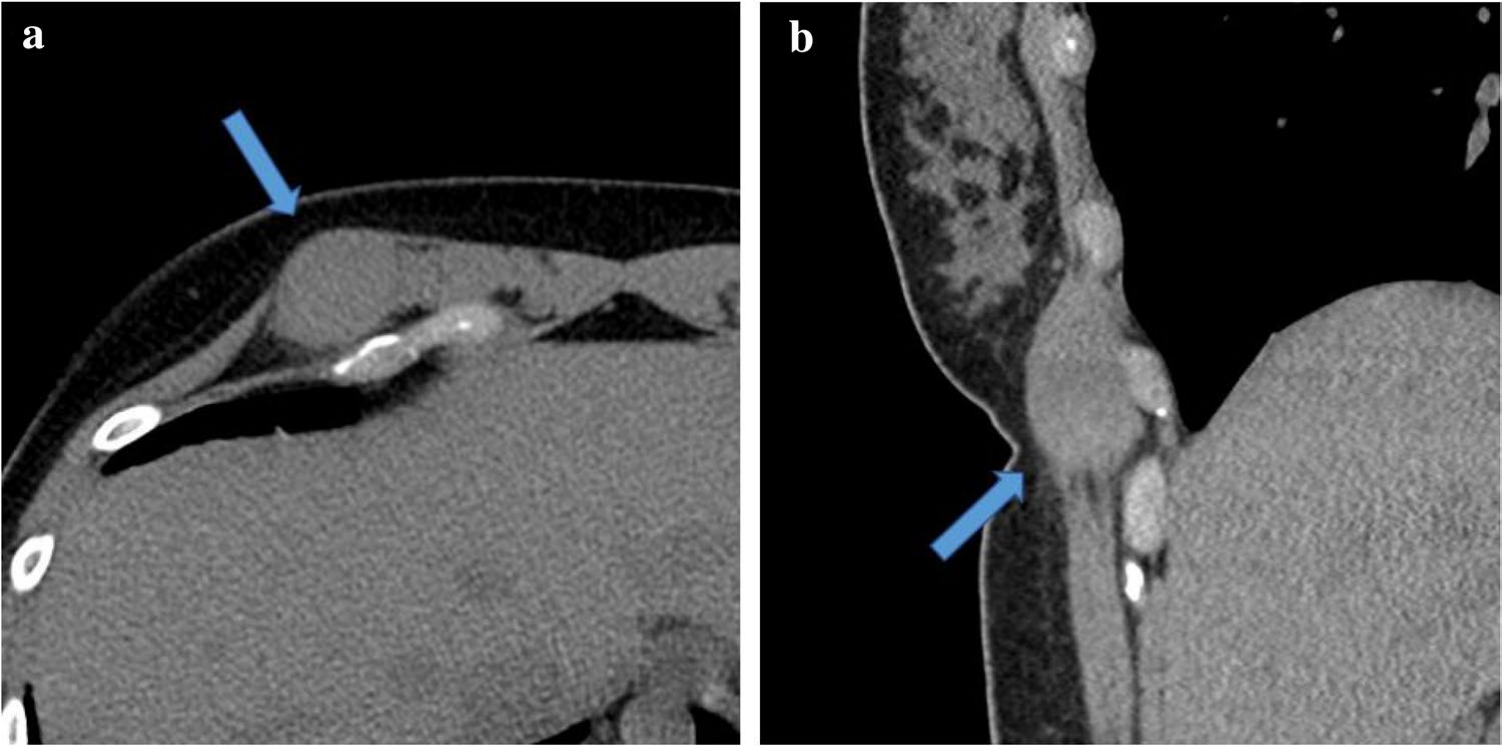
Thirty-five-year-old female with granular cell tumor of the right rectus abdominis muscle. Axial (**a**) and sagittal (**b**) CT chest redemonstrates the right upper abdominal wall mass arising from the superior right rectus abdominis muscle, without evidence of metastatic disease in the thorax (not shown). The mass is iso-attenuating to the surrounding abdominal wall musculature. There is very mild peritumoral fat stranding

The right upper abdominal wall mass was biopsied percutaneously under ultrasound guidance (Fig. 3). Pathology revealed features of a granular cell tumor. Following interdisciplinary discussion at tumor board, the decision was made to proceed with surgical excision for a definitive diagnosis. Preoperative CT scan of the chest did not show any metastatic disease (Fig. 4).

**Fig. 5:**
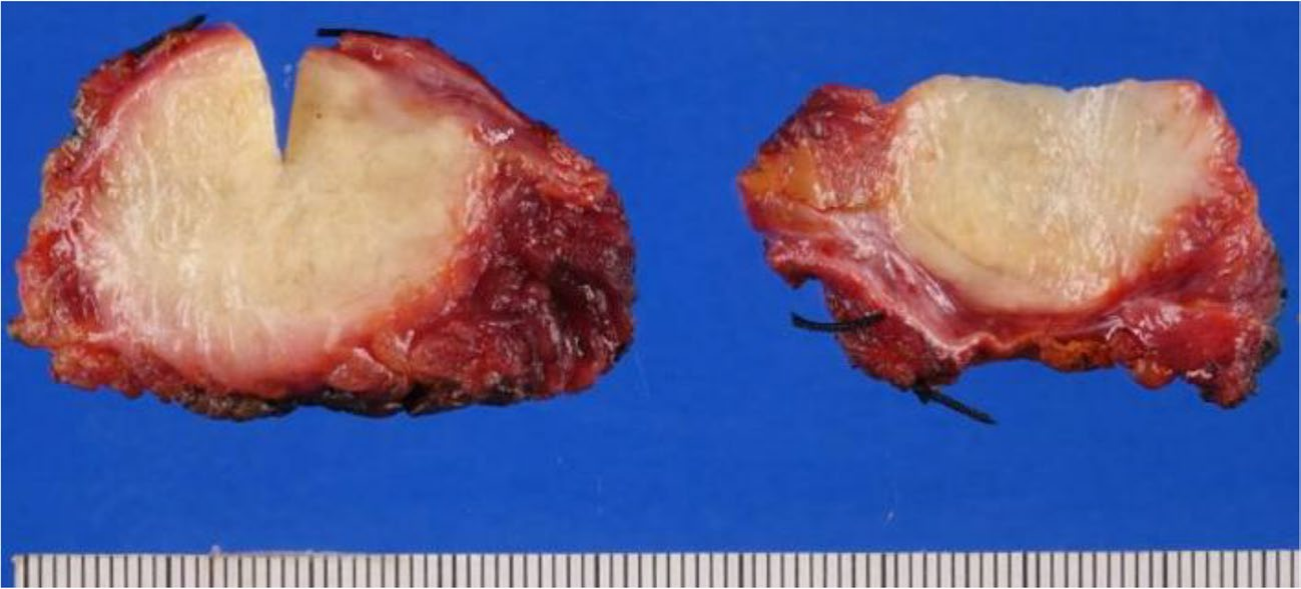
Gross image of tumor

**Fig. 6:**
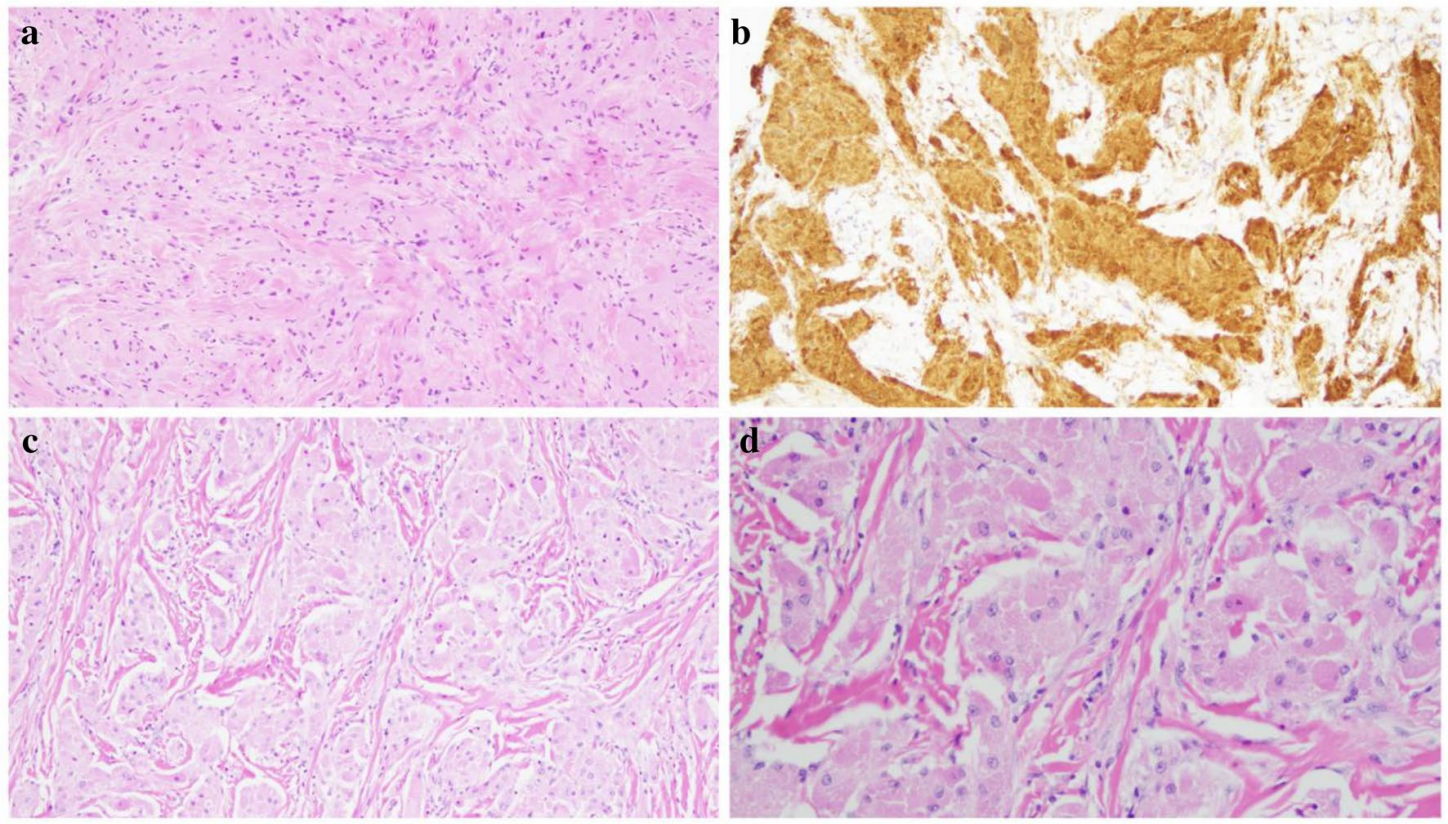
Tumor histology: Initial mass biopsy revealing large cells with eosinophilic, granular cytoplasm (**a**) which stain positive for S100 (**b**). The resection specimen showed a poorly defined mass composed of sheets of cells or nests/ribbons separated by thin collagenous bands (**c**) with more prominent eosinophilic cytoplasm with coarse granules (**d**). **a-c** × 100 magnification, **d** × 200 magnification

**Fig. 7:**
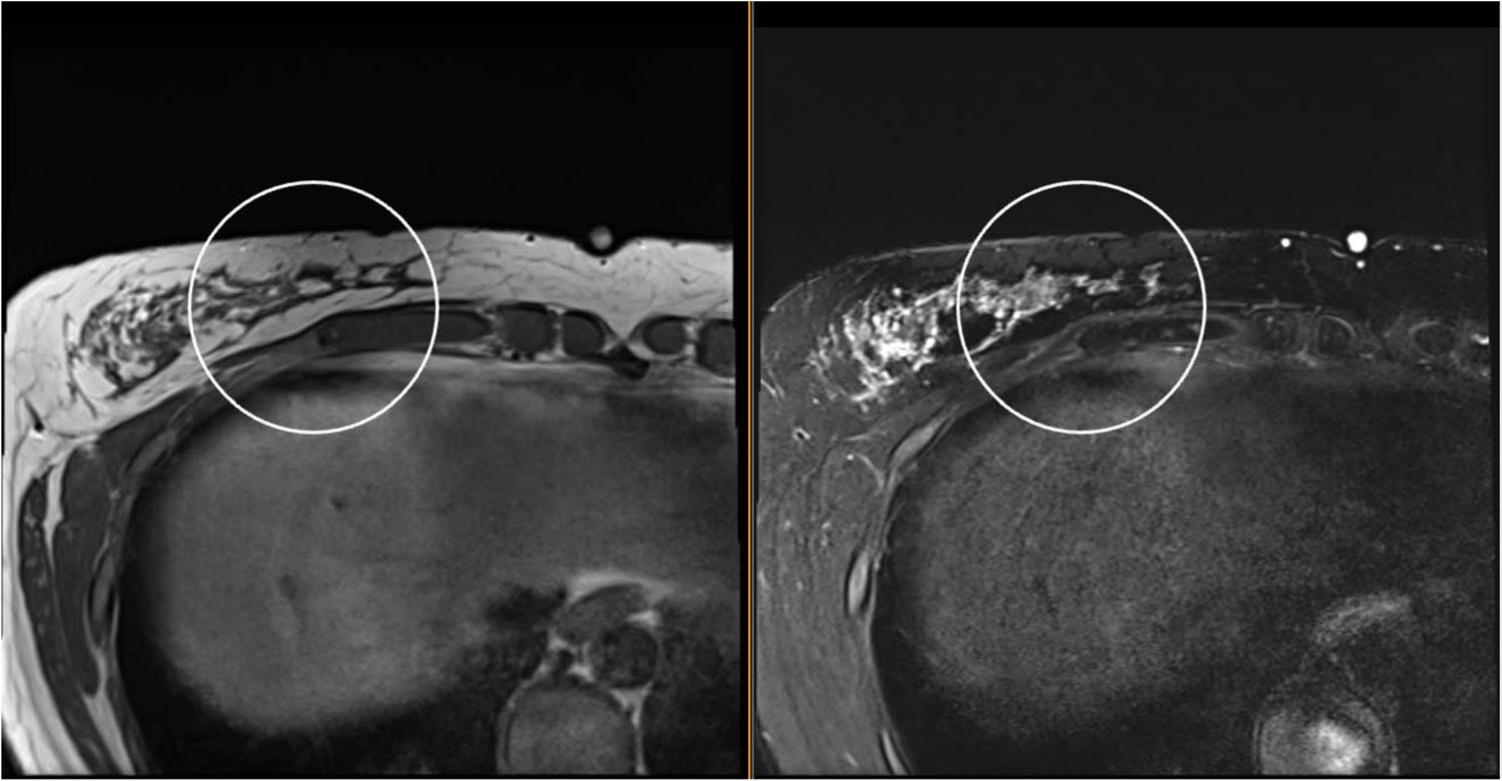
Follow-up surveillance MRI of the chest without contrast approximately 4 months after radical resection of the right chest wall mass shows no mass or signal abnormality to suggest residual or recurrent disease (circle denotes area of previous mass)

The patient underwent radical resection of the right upper abdominal wall tumor. Histopathology confirmed the diagnosis of a low-grade abdominal wall granular cell tumor (Figs. 5 and 6). The tumor stained positive for S100, indicating neuroectodermal origin. The tumor was infiltrative into surrounding tissue, which is typical for this entity. The resection margins appeared negative; however, tumor cells were present within 1 mm of the deep margin. Surveillance MR imaging of the chest wall at 4 months post-resection revealed no evidence of residual or recurrent mass (Fig. 7).

The patient is currently doing well post-resection and will continue to receive surveillance imaging of the chest wall due to narrow surgical margins.

## Discussion

Granular cell tumors (GrCT) are rare soft tissue neoplasms derived from Schwann cells [6]. Most reported cases of GrCT involve the oral cavity, with the tongue being the most common site [14, 15]. However, GrCT can occur in a variety of anatomical locations, including the skin, subcutaneous tissue, breast, and esophagus [15]. GrCTs typically present as small, firm, painless, and slow-growing subcutaneous nodules. GrCT are frequently benign tumors; however, up to 2% of cases present with malignant features [13, 16]. Benign GrCT in the abdominal wall is an extremely rare site of presentation, and to the best of our knowledge, there are only 12 cases of benign GrCT reported in the medical literature to date, which are summarized in Table 1 [1–12].

Initial diagnostic workup with ultrasound allows for assessment of size and extent of the tumors [17]. MRI is the preferred imaging modality to further characterize these soft tissue masses [17, 18]. On MRI, benign GrCTs are isointense to hyperintense to the adjacent muscle on T1 images, and on T2 images, they are isointense to adjacent muscle or suppressed fat and may demonstrate a peripheral rim of hyperintensity [18]. Benign GrCTs are typically oval or round, smaller in size, and superficially located [18]. Few cases of malignant GrCTs have been reported to demonstrate invasion of adjacent structures, large size, and intermediate and low signal intensity on both T1- and T2-weighted sequences [18–20]. More commonly though, GrCT tends to have a non-aggressive appearance, as seen in our patient. Tissue sampling is required to confirm diagnosis and guide treatment. CT may be performed to rule out metastatic disease.

**Fig. 8:**
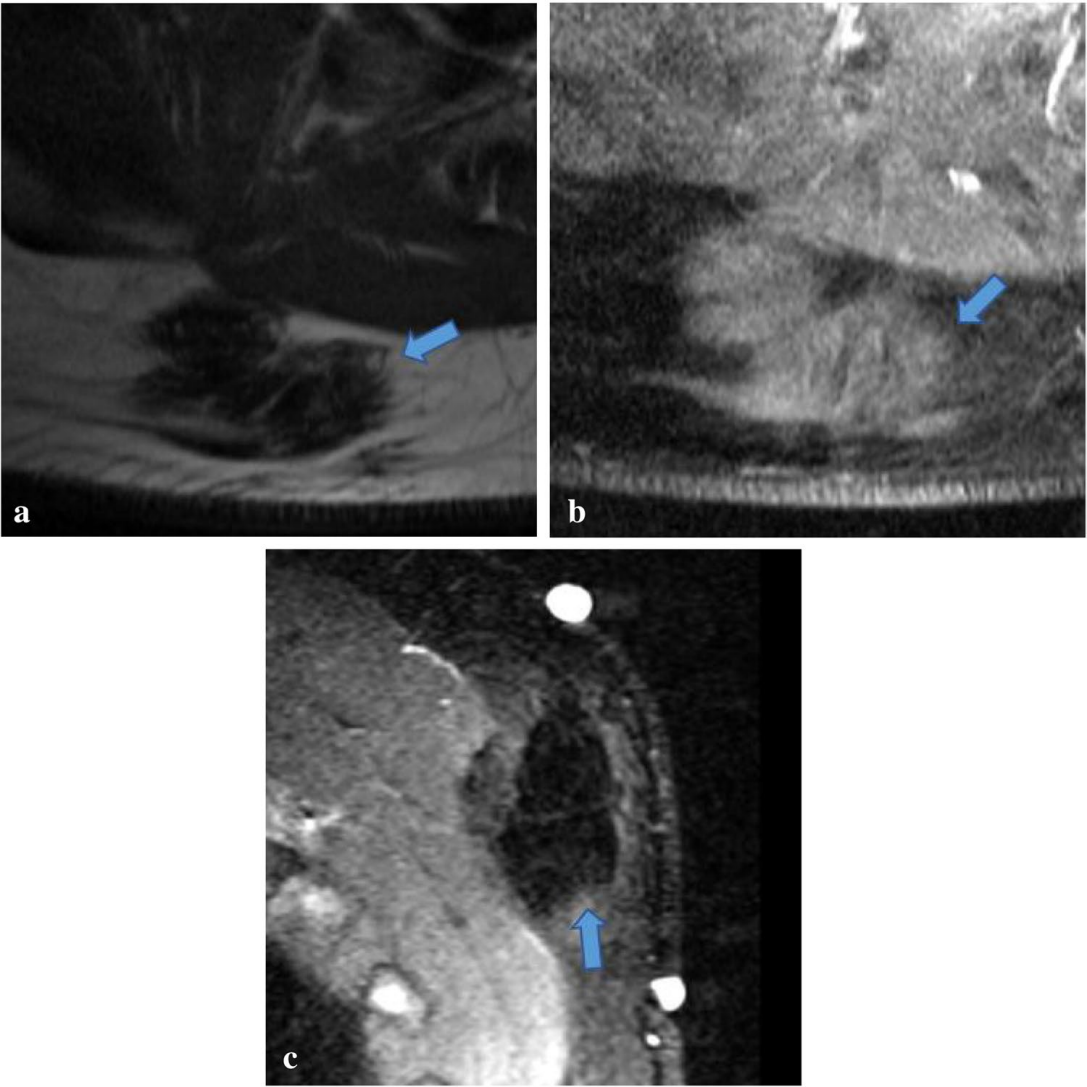
Seventeen-year-old male with history of Gardner’s syndrome. Axial T1 pre-contrast (**a**), axial T1 fat-saturated postcontrast (**b**), and sagittal IR (**c**) images demonstrate an enhancing subcutaneous mass of the upper back (arrow) that is T1-isointense and T2-hypointense to muscle, compatible with desmoid tumor

**Fig. 9:**
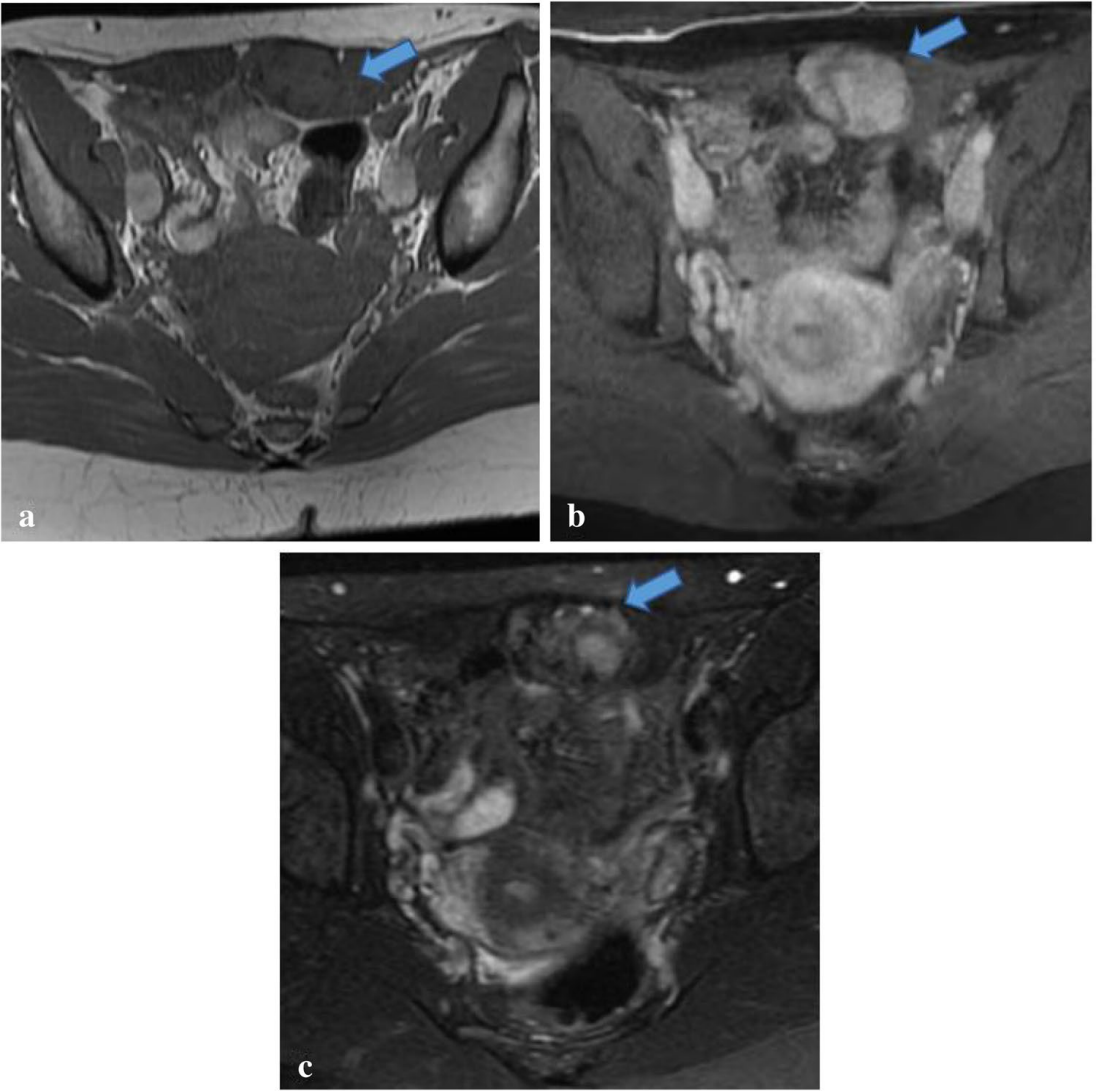
Forty-six-year-old female with desmoid tumor of the left rectus abdominis muscle. Axial T1 (**a**), postcontrast axial T1 fat-suppressed (**b**), and axial T2 fat-suppressed (**c**) images demonstrate an enhancing, heterogeneously T2-hyperintense mass located within the left rectus abdominis muscle (arrow), consistent with desmoid tumor

**Fig. 10:**
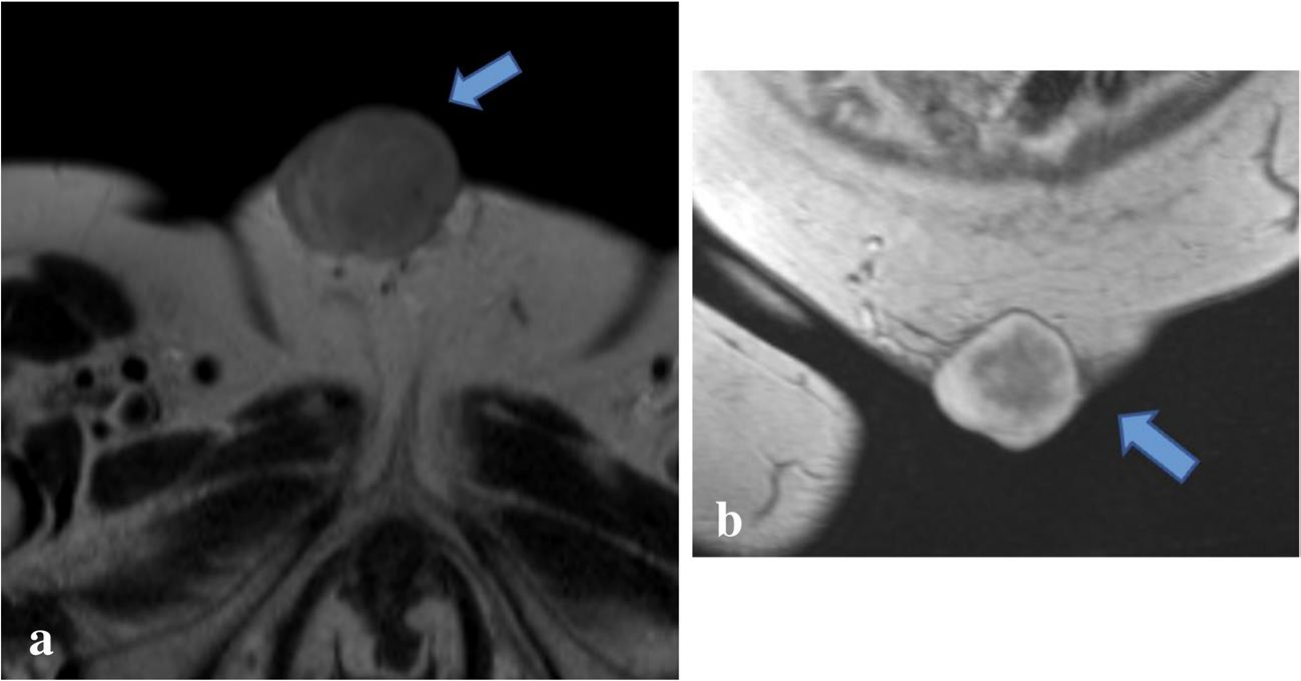
Seventy-eight-year-old female with dermatofibrosarcoma protuberans of the mons pubis. Axial T2 (**a**) and postcontrast coronal T1 (**b**) MRI images demonstrate an enhancing lobulated T2-intermediate signal mass centered at the skin of the mons pubis, measuring 4.5 × 4.1 cm. This tumor was resected and pathology-proven to be dermatofibrosarcoma protuberans, a rare sarcoma

**Fig. 11:**
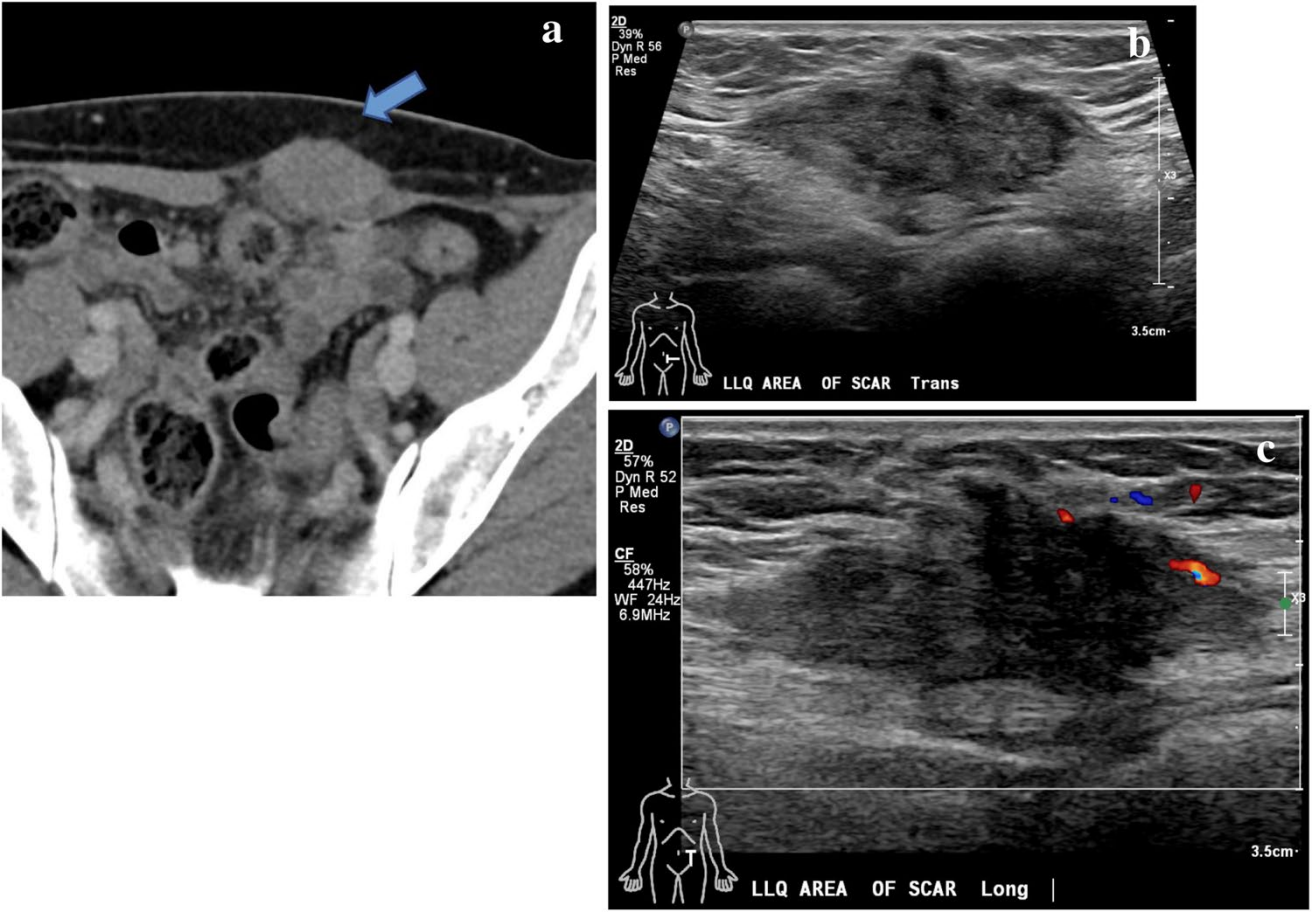
Thirty-six-year-old female with history of Cesarean section and an endometrioma implant of the abdominal wall. Postcontrast axial CT (**a**) of the pelvis demonstrates a homogeneously enhancing oval mass within the left rectus abdominis muscle. Corresponding targeted grayscale (**b**) and Doppler (**c**) ultrasound demonstrates an avascular, heterogeneously hypoechoic soft tissue mass with irregular margins measuring 4.6 cm along the site of Cesarean section scar

**Fig. 12:**
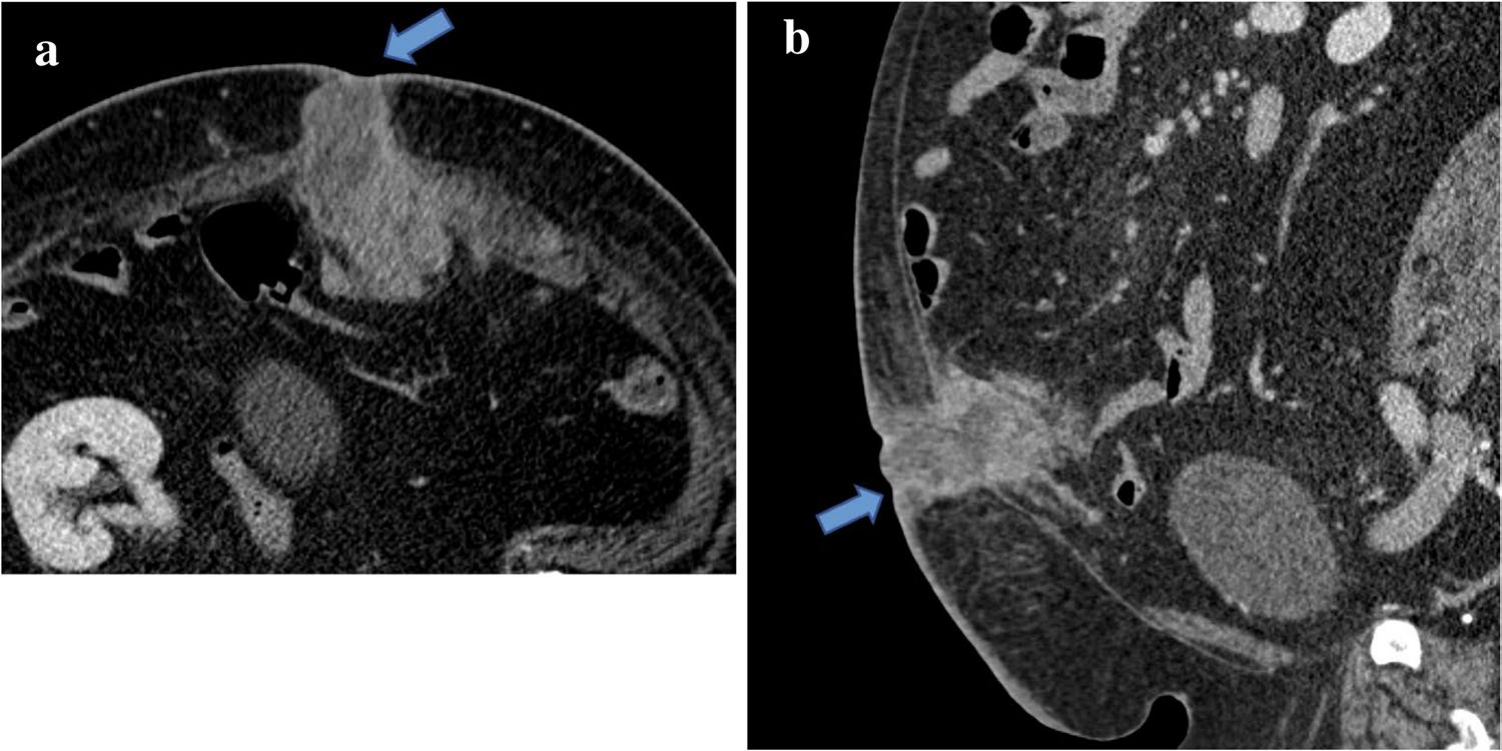
Seventy-one-year-old male with history of hepatocellular carcinoma and biopsy-proven umbilical abdominal wall metastasis. Postcontrast axial (**a**) and sagittal (**b**) CT of the abdomen demonstrates a heterogeneously enhancing mass at the anterior abdomen, with portions that involve the periumbilical abdominal wall and portions that are intraperitoneal and inseparable from small bowel

Because GrCT of the abdominal wall shares imaging features with many other soft tissue tumors including desmoid tumor, sarcoma, endometrioma, and metastasis [21], the diagnosis of GrCT can be difficult to make with respect to the imaging findings alone. For example, on imaging desmoid tumors typically manifest as a soft tissue mass with variable attenuation on CT and variable signal intensities on T1- and T2-weighted MR images with variable enhancement (Figs. 8 and 9) [22]. Sarcomas also often demonstrate heterogeneous attenuation on CT, and varying degrees of necrosis, hemorrhage, or enhancing solid portions may be present (Fig. 10)[21]. Endometrioma implants can be located along a site of surgical scarring within the body wall, and they appear as enhancing nodules on CT; on ultrasound, they typically present as solid hypoechoic nodules with scattered vascularity (Fig. 11) [21]. On MRI, endometrioma implants are iso- to mildly hyperintense relative to skeletal muscle on T1 and T2 images [21]***.*** Metastases to the abdominal wall are also a heterogenous entity, demonstrating variable enhancement and attenuation on CT and variable signal characteristics on MRI (Fig. 12) [21]. Therefore, it is difficult to differentiate GrCT from desmoid tumor and many other differential considerations based solely on imaging. In our patient, we observed a mass that was isoattenuating to muscle on noncontrast CT, T1 iso- to hypointense and T2-isointense to muscle on MRI, with mild, heterogenenous enhancement on postcontrast T1 fat-saturated imaging, which was initially thought to be a desmoid tumor due to its clinical presentation, location, and imaging characteristics. The lesion was overall non-aggressive appearing with mild surrounding soft tissue stranding on CT. Surgical resection and histopathology were ultimately required for definitive diagnosis.

Histologically, GrCT are characterized by large cells with eosinophilic, granular cytoplasm [6]. Diagnosis of GrCT is rendered through a combination of granular cell morphology and positive S100 immunostaining indicating neuroectodermal cell origin. Most GrCT are benign; however up to 2% are malignant [20]. GrCT are often classified as benign, atypical, or malignant based on the histopathologic Fanburg-Smith criteria [13, 16]. These histologic criteria include necrosis, spindling, high nuclear-to-cytoplasmic ratio, increased mitotic activity, and nuclear pleomorphism [13]. A GrCT is considered malignant if 3 or more of these features are identified, atypical if 1 or 2 are identified, and benign if no features are met [13]. Size criteria alone is insufficient to differentiate between benign and malignant lesions [20]. Recently, the molecular driver of disease for GrCTs has been elucidated. Loss of function mutations in the genes related to the vacuolar H + -ATPase (V-ATPase) complex are highly associated with GrCTs [23, 24], and these mutations are rarely seen in other malignancies. Nevertheless, genetic testing is not typically required for making the histopathologic diagnosis of GrCT.

The treatment for benign GrCT is wide local resection to negative margins. The long-term prognosis is excellent, and the recurrence rate is low.

In conclusion, we described the case of an abdominal wall GrCT mimicking the clinical presentation of desmoid tumor in a young, recently postpartum 35-year-old woman. A review of the diagnostic workup of GrCT imaging and histopathologic findings was discussed. Since the most common diagnosis for a painless, solid mass contiguous with abdominal wall musculature with nonaggressive imaging features in a peripartum young woman is a desmoid tumor [25], the final histological diagnosis of benign GrCT in this case was quite unusual. Although rare, abdominal wall GrCT should be considered in the differential diagnosis when working up similar patients with firm, soft tissue mass of the abdominal wall.

## Data Availability

The data supporting the findings presented in this case report are available within the manuscript and supplementary tables and figures, with patient identifiers removed to protect confidentiality.
